# Life Cycle Assessment of Thermoactivated Recycled Cement Production

**DOI:** 10.3390/ma15196766

**Published:** 2022-09-29

**Authors:** Sofia Real, Vitor Sousa, Inês Meireles, José Alexandre Bogas, Ana Carriço

**Affiliations:** 1 Civil Engineering Research and Innovation for Sustainability (CERIS), Instituto Superior Técnico, Universidade de Lisboa, Av. Rovisco Pais, 1049-001 Lisbon, Portugal; 2 Research Center for Risks and Sustainability in Construction (RISCO), Departamento de Engenharia Civil, Universidade de Aveiro, 3810-193 Aveiro, Portugal

**Keywords:** life cycle assessment, thermoactivated recycled cement, recycled cement paste liberation, recycled cement paste separation

## Abstract

The urgent need to tackle the effects of global warming has led to a worldwide compromise and ever-more demanding regulations. In this respect, as an important greenhouse gas emitter, the cement industry has to implement major changes in its production processes to achieve future goals. In this perspective, low-carbon eco-efficient cement, such as the thermoactivated recycled cement from concrete waste (RCC), seem to be a promising alternative to current carbon-intensive binders, such as ordinary Portland cement (OPC). This study aimed to demonstrate the potential contribution of RCC to the reduction in the environmental impacts of the cement industry, by means of a comparative life cycle assessment of three production methods of this binder (wet (WM), dry (DM) and air clean (ACM) methods) and OPC. Overall, RCC WM did not turn out to be a good alternative to OPC, essentially owing to the amount of fuel and electricity required for washing and drying the particles before the magnetic separation. On the other hand, RCC DM and RCC ACM proved to be promising alternatives to RCC WM and OPC, with a relevant reduction in all impact categories.

## 1. Introduction

The effects of global warming are being felt worldwide and the cause is attributed to the greenhouse effect of gases stemming from natural and human activities [1,2]. Carbon dioxide (CO_2_) emissions have been pointed out as one of the major contributors to the greenhouse effect and, consequently, global warming [1,2]. In this respect, the urgent need to tackle this problem has led to a worldwide compromise and ever more demanding regulations. The Kyoto protocol [3] strived for member nations to reduce greenhouse gas emissions (GGE) from various economic activities by about 5.2% compared to 1990, between 2008 and 2012. In 2014, EU countries adopted the 2030 climate and energy framework [4], which includes the pursuit of the reduction of at least 40% of GGE by 2030, compared to 1990. These objectives were bolstered by the Paris agreement, in 2015 [5], with the commitment for the reduction of 60–80% of GGE by 2050, compared to 1990.

In parallel, human activities generate large amounts of residues, which also have a very relevant contribution to environmental degradation. To counteract this environmental issue, the European Waste Framework Directive 2008/98/CE [6] established the target of reusing/recycling 50% of the generated waste by 2020. In Portugal, the law-decree 102-D/2020 [7] strived to stimulate the adoption of sustainable production and consumption models with the purpose of further reducing residue production.

In the construction industry context, cement is currently the most used building material [8], with over 4 billion tonnes produced yearly [9]. Aside from consuming about 1.6 tons of raw material [10] and over 3600 MJ of thermal energy from fossil fuels [11,12], the production of a ton by the binder releases about 750–800 kg of CO_2_ into the atmosphere [13]. Most of these emissions, about 85%, are related to the sintering process, of which around 60% are credited to the decarbonation of limestone [14,15,16], and the remaining 40% to the fossil fuel burned to generate the thermal energy required for the production [9]. Actually, cement production constitutes about 80–90% of the CO_2_ emissions in concrete production [17,18]. On the other hand, construction and demolition waste constitute about half of the industrial waste, of which 65% is concrete waste [19]. According to Neville and Aïtcin [20], high-performance concrete typically has about 400–550 kg/m^3^ in its composition. Thus, cement is recognisably one of the main contributors to the environmental footprint of the construction industry.

Overall, the total CO_2_ emissions related to cement production represent about 8% of the annual global CO_2_ emissions [9,21], being estimated that the cement industry could reduce about 18% of the direct emissions by 2050 by taking the proper actions [13]. At the same time, the European Directive 2008/98/CE [6] already requires that 70% of the construction and demolition waste should be recycled or reused. Recently, Portuguese law-decree 102-D/2020 [7] established prevention and reduction goals to mitigate the human health and environmental impacts of residue production, having imposed a 5% and 10% reduction in the amount of non-urban residues per PIB unit, including those from the construction sector, by 2025 and 2030, compared to 2018 values. It also requires that the materials used in public construction works are composed of at least 10% recycled materials.

The cement industry has already improved significantly the energy efficiency of the production process (e.g., evolution from wet to dry production process), reducing the carbon content and overall environmental impact of the fuels used (e.g., incorporation of waste in the fuel mix) and incorporated a wide variety of low carbon additives directly into the cement or in the composition of the concretes (e.g., fly ashes, ground granulated blast furnace slag). Still, to meet the carbon neutrality goal, the cement industry has to implement major changes in its production processes [9]. To that end, WBCSD and IEA [13] suggest the following main groups of measures for the reduction in CO_2_ emissions in the cement production process: (i) the implementation of new technologies and equipment modernisation for higher energetic efficiency; (ii) alternative fuel usage; (iii) the substitution of carbon-intensive cement with low carbon cementitious materials; and (iv) CO_2_ capture before being released to the atmosphere and adequate storage or use. The alternatives already existing or being explored in each of the groups of are multiple. Within the substitution of carbon-intensive cement with low-carbon cementitious materials, it is possible to distinguish two groups of options: (i) use of decarbonised raw material for cement production and (ii) exploring alternative low-carbon binders or the replacement of cement by low-carbon admixtures. Some of the low-carbon cement being explored include belite-rich cement, geopolymers, alkali-activated cement, calcium aluminate cement, low-temperature or modified clinkers, blended cement with alternative supplementary cementitious materials and emerging non-Portland cement clinker-based binders [22,23].

From this perspective, low-carbon eco-efficient cement, such as thermoactivated recycled cement (RC), seem to be a promising alternative to current carbon-intensive binders, such as ordinary Portland cement (OPC) [24,25,26,27,28,29]. In addition to the benefits for the cement industry, this option has additional benefits of contributing to meeting the construction and demolition waste and circular economy targets by promoting recycling instead of downcycling.

There is extensive literature on LCA studies applied to cement [10,30,31,32,33,34], since it is a highly consumed material with significant environmental impacts. However, environmental assessments of thermoactivated recycled cement production using this new process for separating the cement paste from the aggregates are limited to the energy and carbon balances developed by Sousa and Bogas [35] and Sousa et al. [36].

This study aims to demonstrate the potential contribution of thermoactivated recycled cement to the reduction in environmental impacts of the cement industry, by means of a comparative life cycle assessment (LCA) of different production methods of this binder and OPC.

## 2. Basic Description of Thermoactivated Recycled Cement

The production process of thermoactivated recycled cement from concrete waste (RCC) considered in this study was developed by the authors [37,38] and its characterisation and technical viability for application in building materials have been demonstrated in a laboratory [28,29]. In previous studies by the authors [28,29], concrete with RCC has been found to be able to present comparable mechanical and durability performance to that of OPC concrete of equal water/binder ratio.

The production process starts with the most challenging phase, which is the liberation and magnetic separation of the hydrated cement paste particles from the aggregates in the concrete waste. The first stage of this patented process [38] comprises the mechanical crushing, grinding and milling of the concrete waste, in order to obtain fine liberated paste particles and aggregate particles under 1 mm. The granulometric fractions were established taking into account a previous extensive experimental work of the authors [37], which was the base of an innovative separation method that is under patent [38]. Then, the liberated particles are divided into three main granulometric fractions (150–250 μm, 250–500 μm and 500–1000 μm) which, according to laboratory results, comprise about 80.5% (12.3%, 29.9% and 38.3%, respectively) of the liberated particles and are the most suitable for the magnetic separation stage [37]. In order to maximise the efficiency of the magnetic separation in laboratory conditions, the liberated particles undergo a washing and drying stage (Wet method (WM)), to eliminate the remaining ultrafine particles that could hinder the separation process [37]. This stage results in a minor material loss (<1%). Afterwards, the clean liberated particles pass through a magnetic roll with 1.4 T, which separates the magnetic (cement paste) from the non-magnetic particles (aggregates). This process is repeated for the obtained magnetic particles to improve the quality of the resulting material. At the end of this stage, high-quality recycled cement paste particles and recycled sand are obtained. The latter may be employed for various purposes, also with potential economic, social and environmental benefits compared to natural or artificial sand, but this is outside of the scope of the present research effort.

The magnetic recycled cement particles obtained from the two granulometric fractions that yield the better quality recycled cement, 150–250 μm and 250–500 μm, correspond to about 20% and 16.5% of the clean-liberated particles with about 89% and 73.6% purity, respectively [37]. For the production of RCC, the obtained recycled cement paste particles undergo further milling to obtain particles under 150 μm. The final phase consists of the thermal activation of the fine recycled cement paste particles at 650 °C, which allow the recovery of the rehydration capacity of the cement without significant decarbonation [24]. In laboratory conditions, the thermal treatment entails heating at 10 °C/min up to treatment temperature, followed by a residence period of 3 h and cooling inside the kiln until ambient temperature is reached [24,37].

Recently, after estimating the contribution towards energy consumption and CO_2_ emissions of each stage of the recycled cement production process [35], small variations to this production process were tested. These variations were aimed at eliminating the need for washing and drying the material prior to the magnetic separation. As such, the separation process was performed in non-washed liberated particles (Dry method (DM)). Though this processing method was simpler and required less resources, for 150–250 μm and 250–500 μm fractions, the purity of the obtained magnetic material was only about 63% and 73% of cement paste content, and its yield was about 30% and 73%, respectively. The relevant purity reduction in the finer fraction in the DM led to the development of the Air clean method (ACM), which consisted of the cleaning of the recycled cement particles with compressed air before the separation process. Despite requiring an extra step compared to the RCC DM, the RCC ACM resulted in about 19.9% and 16.3% of magnetic particles with 93% and 76% purity, for 150–250 μm and 250–500 μm fractions, respectively.

Moreover, different thermal activation residence periods were tested for the production of RCC in laboratory conditions. Minor variability of the properties of RC was found for residence periods between 1 and 5 h, suggesting that the residence period could be reduced to at least as little as 1 h. It is possible that, in industrial conditions, the residence period could be lower than 1 h, since the length of the thermal treatment is, in a significant part, owing to the technical limitations of the laboratory oven. In particular, the ventilation of the water vapour to prevent its reaction with the reactivated cement. The dehydration and depolymerisation of the hydrated cement paste result in a 20–25% weight reduction [24,37].

## 3. Methodology

In this study, the *SimaPro 9* software was used to assess the environmental performance of RCC compared to OPC using a “cradle-to-gate” approach. The LCA followed the recommendations provided by ISO 14040 [39] and ISO 14044 [40], as well as by ISO 14025 [41] and EN 15804 [42]. The declared unit was considered to be 1 kg of the produced binder.

The life-cycle inventory was carried out according to the guidelines of EN 15804 [42] for the product stage, including materials, fuels and electricity, as well as air and water emissions and waste treatment. The OPC production is one of the processes already available on the *Ecoinvent 3* database. This document also served as a basis for performing the LCA of RCC. Extrapolations based on analogy supported by the experimental data, for the stages that are common or similar between RCC and OPC production, complemented with the literature information, for the stages that are distinct between RCC and OPC production, were used to develop a product document for RCC.

The LCA was performed resorting to the *CML-IA baseline* method, usually adopted for the construction industry, which includes the various environmental impact categories covered by EN 15804 [42], namely the potential for abiotic depletion (materials and fuels), global warming, ozone layer depletion, human toxicity, freshwater aquatic ecotoxicity, marine aquatic ecotoxicity, terrestrial ecotoxicity, photochemical oxidation, acidification and eutrophication.

## 4. Life-Cycle Inventory

### 4.1. Ordinary Portland Cement

The life-cycle assessment of the production of OPC has been extensively addressed in the literature [10,30,31,32,33,34]. Thus, the *Ecoinvent 3* database provides several examples of this process. In this study, the life-cycle assessment of the production of OPC was only performed for comparison purposes and the process of *Cement, Portland Europe without Switzerland* was chosen. This regional restriction was established, due to the context in which the RCC is being developed and to the overall goal of aligning it with the European goals for sustainable development. This *Ecoinvent 3* database process includes the whole production process, namely natural resources extraction, clinker calcination and mixture with gypsum, ending in the cement mill.

### 4.2. Thermoactivated Recycled Cement from Concrete Waste

**Figure 1:**
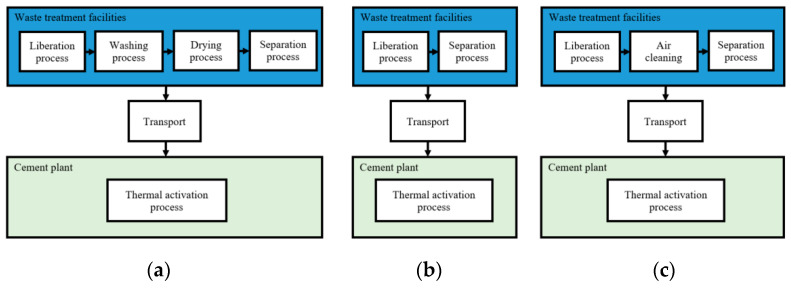
Schematic representation of the different production phases of RCC production through: (**a**) Wet method; (**b**) Dry method; (**c**) Air clean method.

For the LCA of RCC, three production methods were considered, depending on the tasks required during the separation process, specifically the cleaning of the material prior to the magnetic separation. The background of RCC production was presented in Section 2 and a summary of the production details of the different methods is displayed in Figure 1.

After the particle size reduction process and before the thermal activation of the recycled cement particles at 650 °C for under 1 h, in the wet method, the concrete waste particles are washed and dried before magnetic separation; in the dry method, the concrete waste particles are not washed and dried before magnetic separation; and in the air clean method, the concrete waste particles are air cleaned before magnetic separation.

The production of the RCC process was partially based on the *Ecoinvent 3* database *Clinker Europe without Switzerland* process, which is also a part of the *Cement, Portland Europe without Switzerland* process. This process essentially accounts for the part of the cement process that is similar to RCC production, given that RCC is not mixed with gypsum nor other additional components. For the creation of the inventory for its production process, RCC was assumed to be industrially implemented by resorting to two types of existing facilities, namely: the construction and demolition of waste treatment facilities, where the concrete waste has already been separated from steel reinforcement and other contaminating materials, and the liberation and separation stages would take place; the cement plant, where the thermal activation of the RCC would take place. The separated recycled cement particles are transported between the waste treatment facilities and the cement plant by truck. This transport was modelled considering a 50 km distance between the waste treatment facilities and the cement plant (back and forward—100 km) and using the Ecoinvent 3 database Transport, freight, lorry, unspecified process.

Additionally, the following assumptions/approximations were also made: (i) no natural raw materials are used in the production of RCC, except for water in the Wet method—the amount of water required to wash the liberated concrete waste particles in the Wet method was considered to be about 2 m^3^/ton of waste material, using the *Ecoinvent 3* database *Water, unspecified natural origin* process; (ii) for the liberation and separation process of RCC, the amount of electricity required for crushing, grinding, washing, drying and separating the concrete waste particles was estimated for each production method using the approach developed in Sousa et al. [36], using the *Ecoinvent 3* database *Electricity, medium voltage* process; (iii) to determine the amount of fuel required for the thermal treatment phases of RCC, a treatment temperature of 650 °C for a residence period identical to that of clinker calcination was considered (Section 2), as well as the varying weight loss during thermal treatment (see Sousa and Bogas [35] for more details); and (iv) for the Wet method, the amount of fuel needed to dry the washed concrete waste particles was estimated considering the results obtained by Sousa and Bogas [35].

The ratio between the fuel consumption of the RCC and OPC was used to define a conversion factor that was applied to the fuel inputs and outputs of *Clinker Europe without Switzerland* process to determine the fuel inputs and outputs of the RCC processes. This implies the assumptions that the majority of the outputs from OPC production, in particular air emissions, resulting from the combustion of fossil fuels [43] and that the thermal reactivation of the recycled cement paste will only release water vapour. The only exception was the CO_2_ emissions from fossil fuels. Given that part of this output stems from the decarbonation of clinker raw materials, which does not occur in RCC, the portion of emissions attributed to this phenomenon (0.534 kg CO_2_/kg clinker) was discounted before applying the conversion factor. Similarly to the *Cement, Portland Europe without Switzerland* process, no packaging was considered for the RCC processes.

## 5. Life Cycle Assessment

As mentioned in Section 3, the LCA was carried out through the analysis of the environmental impacts, divided into 11 categories: abiotic depletion, materials and fuels, global warming, ozone layer depletion, human toxicity, freshwater aquatic ecotoxicity, marine aquatic ecotoxicity, terrestrial ecotoxicity, photochemical oxidation, acidification and eutrophication. Table 1 displays the results of the LCA of the different types of binder, according to each impact category.

### 5.1. Abiotic Depletion

The abiotic depletion potentials (materials and fuels) are essentially associated with the extraction of non-living natural resources [44,45]. As such, the abiotic depletion potentials of OPC are essentially affected by the raw materials (bauxite, calcareous marl, clay, lime and sand) and fuel consumption. Conversely, RCC is produced with recycled concrete waste and does not require the extraction of raw materials. Moreover, taking into account that, except for the transportation between the waste treatment facilities and the cement plant, the fuels are used for heating during thermal treatment and that the production of clinker requires a treatment temperature more than twice as high as that of RCC, the amount of fuel required for RCC thermal treatment is significantly lower than for clinker production. However, the amount of concrete waste that needs to be processed to obtain a unit of RCC is substantially higher than the amount of raw material needed to obtain the same amount of clinker, since the recycled cement paste is only a small fraction of the concrete volume and the separation process is not capable of retrieving all of the recycled cement paste. As a result, the amount of electricity consumed in the liberation and separation stages of RCC production is higher than for processing the raw material for clinker production. More importantly, in the RCC WM, this also implies a substantial consumption of fossil fuels for drying the washed concrete waste particles, prior to the magnetic separation. The RCC production implementation considered herein requires that the separated recycled cement is transported from the construction and demolition of waste treatment facilities to the cement plant, which the clinker does not require, owing to the fact that raw materials for this binder are usually extracted from nearby quarries.

**Figure 2:**
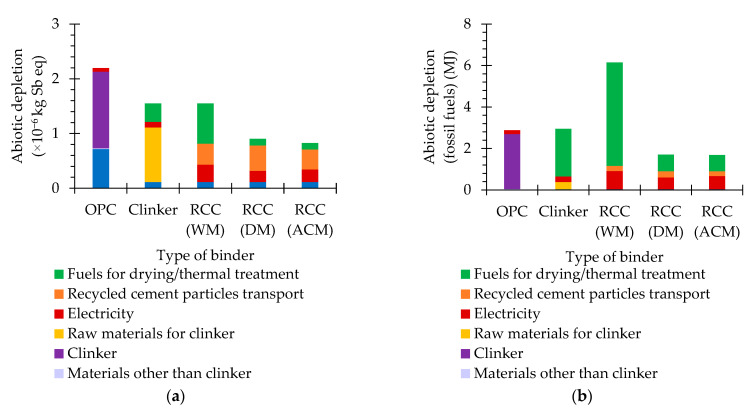
Abiotic depletion potentials of different types of binder: materials (**a**) and fuels (**b**).

Depending on the RCC production method, the abiotic depletion potentials are about 38–71% (materials, Figure 2a, Table 1) and 58–214% (fuels, Figure 2b, Table 1) of those of OPC. The abiotic depletion potentials of RCC DM are roughly 58% (materials, Figure 2a) and 28% (fuels, Figure 2b) of those of RCC WM, due to the fact that the concrete waste particles do not require washing and drying before the separation process, and thus, less electricity and fuel are needed to produce this binder.

Furthermore, the abiotic depletion potentials of RCC ACM are about 92% (materials, Figure 2a) and 99% (fuels, Figure 2b) of those of RCC DM. Despite the fact that RCC ACM requires slightly more electricity than RCC DM, the contribution of the substantially higher purity of the RCC ADM particles is enough to offset the additional electricity consumption on these impact categories. Altogether, the abiotic depletion potentials of both RCC DM and RCC ACM are substantially lower than those of OPC. Nonetheless, though it contributed to the reduction in the material’s abiotic depletion potential, the need for washing and drying during the liberation and separation process, the fossil fuels’ abiotic depletion potential of RCC WM is worse than that of OPC.

### 5.2. Global Warming

The global warming potential is one of the most used categories for assessing the environmental impact of industrial processes [31,46]. This category concerns the effects of greenhouse gas emissions on the atmosphere, which may contribute to temperature increase and affect the ecosystem health, as well as human health [44,45]. Greenhouse gas emissions essentially comprehend CO_2_, methane (CH_4_), nitrous oxide (N_2_O) and other fluorinated gases, where the gross majority is attributed to CO_2_ emissions [43,47,48]. The CO_2_ emissions from OPC production are mainly associated with fuel burning and raw-material decarbonation during clinker production. In RCC production, the latter is essentially avoided, given that the treatment temperature is lower than that required for decarbonation of lime to occur. Furthermore, the amount of fuel burned during the thermal treatment of RCC is significantly lower than that required for clinker production.

**Figure 3:**
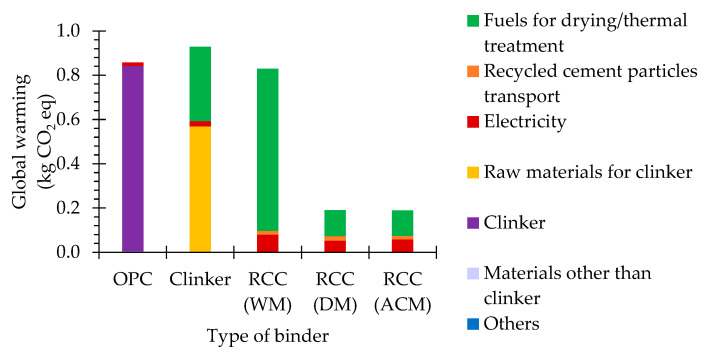
Global warming potential of different types of binder.

Nonetheless, the processing of the concrete waste to retrieve the recycled cement paste requires a substantial amount of electricity, and in RCC WM, also of fuel, which contributes to the global warming potential. In total, the global warming potential of RCC is about 22–92% of that of OPC, depending on the production method (Figure 3, Table 1). As RCC DM and RCC ACM require less electricity and, especially, less fuel, their global warming potential is only close to 23% of that of RCC WM (Figure 3, Table 1). The global warming potential of RCC ACM is similar to that of RCC DM (about 99%).

Overall, RCC DM and RCC ACM have a staggering potential for effectively reducing the global warming potential compared to OPC. Conversely, due to the need for washing and drying the concrete waste before the magnetic separation of the recycled cement paste particles, the contribution of RCC WM to the reduction in the global warming potential is not as relevant as those of RCC DM and RCC ACM.

### 5.3. Ozone Layer Depletion

The ozone layer depletion potential is related to the effects of the reduction in the ozone layer, which may have damaging consequences for human health and ecosystems, as increasing amounts of UV-B radiation get through to the Earth’s surface [44,45].

The ozone layer depletion potential of OPC is basically determined by its clinker content, which, in turn, is essentially influenced by fuel consumption and, less significantly, by raw material and electricity consumption. As RCC requires less fuel for thermal activation than clinker, its contribution to this impact category is not as significant. Moreover, RCC has no raw material consumption (with the exception of the water used for washing the concrete waste particles in the RCC WM). However, as mentioned, due to the liberation and separation process, RCC generally required more electricity, and for RCC WM, more fuel to process the concrete waste than the clinker was needed to process the raw material. Furthermore, the transport of the recycled cement paste particles from the waste treatment facilities to the cement plants also contributes to the ozone layer depletion potential of RCC.

**Figure 4:**
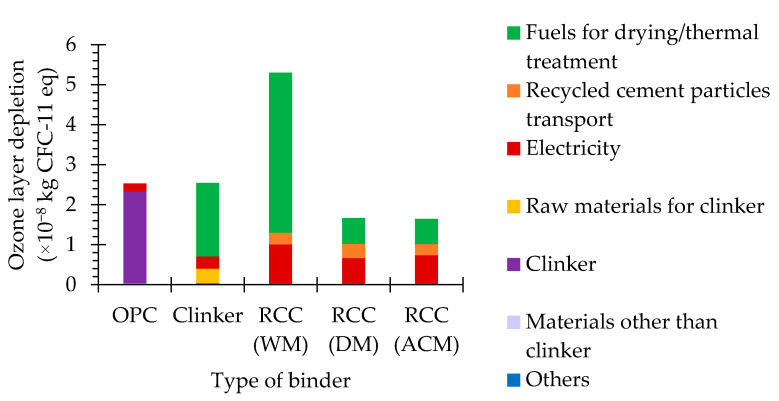
Ozone layer depletion potential of different types of binder.

Overall, the ozone layer depletion potential of RCC ranged from 65% to 210% of that of OPC, depending on the production method (Figure 4, Table 1).

The ozone layer depletion potentials of RCC DM and RCC ACM were roughly 31% of that of RCC WM, due to the fact that RCC WM requires more electricity and fuel during the liberation and separation stages of the concrete-waste processing (Figure 4, Table 1). The ozone layer depletion potential of RCC ACM is comparable to that of RCC DM (about 99%). In sum, RCC DM and RCC ACM have a positive effect on the reduction in the ozone layer depletion potential compared to OPC. However, owing to the need for washing and drying during the liberation and separation process, RCC WM results in a substantial increase in the ozone layer depletion potential compared to OPC.

### 5.4. Human Toxicity and Freshwater Aquatic, Marine Aquatic and Terrestrial Ecotoxicity

The human toxicity and freshwater aquatic, marine aquatic and terrestrial ecotoxicity potentials are related to the effects of toxic substances emitted to air, water and soil on human health and on aquatic and terrestrial ecotoxicity, respectively [44,45].

As previously observed for other impact categories, the human toxicity and freshwater aquatic, marine aquatic and terrestrial ecotoxicity potentials of OPC were mainly affected by its clinker content, which was primarily influenced by raw material, fuel consumption and, to a lesser extent, electricity consumption.

There is no raw material consumption in RCC production and the fuel needs for thermal activation are considerably lower than those of clinker. Therefore, the influence of these parameters on this impact category is not as expressive. On the other hand, RCC production involves a significantly higher amount of electricity, and in RCC WM, of fuel, than that of clinker.

**Figure 5:**
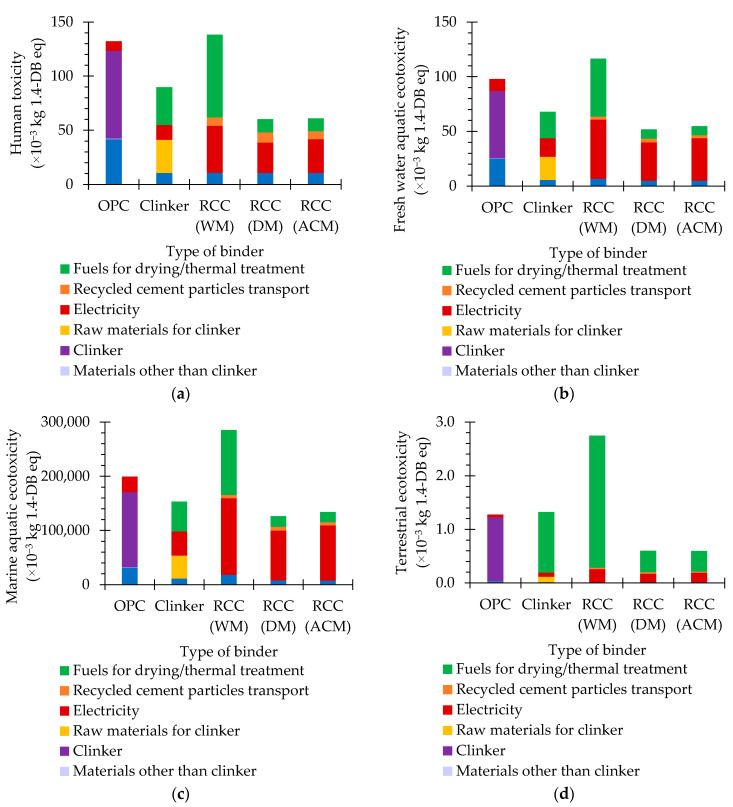
Human toxicity (**a**) and freshwater aquatic (**b**), marine aquatic (**c**) and terrestrial ecotoxicity (**d**) potentials of different types of binder.

Depending on the liberation and separation method, the human toxicity and freshwater aquatic, marine aquatic and terrestrial ecotoxicity potentials of RCC vary from 46% to 105%, 53% to 119%, 63% to 143% and 47% to 215% of those of OPC, respectively (Figure 5, Table 1). Given the higher electricity and fuel need of RCC WM, the human toxicity and freshwater aquatic, marine aquatic and terrestrial ecotoxicity potentials of RCC DM and RCC ACM are about 44%, 45–47%, 44–47% and 22% of those of RCC WM, respectively (Figure 5, Table 1). Additionally, due to the fact that RCC ACM required more electricity than RCC DM, the human toxicity and freshwater aquatic, marine aquatic and terrestrial ecotoxicity potentials of RCC ACM are estimated to be 101%, 105%, 106% and 100% of those of RCC DM, respectively (Figure 5, Table 1).

In general, RCC DM and RCC ACM have a beneficial effect on the reduction in human toxicity and freshwater aquatic, marine aquatic and terrestrial ecotoxicity potentials compared to OPC. On the other hand, RCC WM did not have a positive effect on these potentials. It is important to mention that the electric energy estimates for RCC production are conservative and were partially determined by analogy with the OPC production, following the approach developed in Sousa et al. [36], using a reference determined for RCC production with a considerably higher electricity consumption (155 kWh/t clinker) than the value in the *Ecoinvent 3* database (58 kWh/t clinker) for the production process of clinker.

### 5.5. Photochemical Oxidation, Acidification and Eutrophication

The photochemical-oxidation potential concerns the formation of ozone through the exposure of some air pollutants to sunlight, which affects human health and ecosystems [44,45]. Essentially, the photochemical oxidation of volatile organic compounds (VOC) and carbon monoxide (CO) occurs in the presence of nitrous oxides (NOx), under ultraviolet light, forming ozone [45]. The acidification potential pertains to the effects of acidifying pollutants on soil, water, organisms, ecosystems and materials [44,45]. These acidifying pollutants include gas emissions from fossil fuel combustion, namely sulphur dioxide (SO_2_), NOx and ammonia gas (NHx) [45]. The eutrophication potential involves the effects of the disproportionate levels of macro-nutrients (namely nitrogen (N) and phosphorous (P)) in the environment, owed to nutrient emissions to air, water and soil [44,45]. The main consequences of this nutrient enrichment are potential alterations in species composition and high-biomass production in the ecosystems [45].

**Figure 6:**
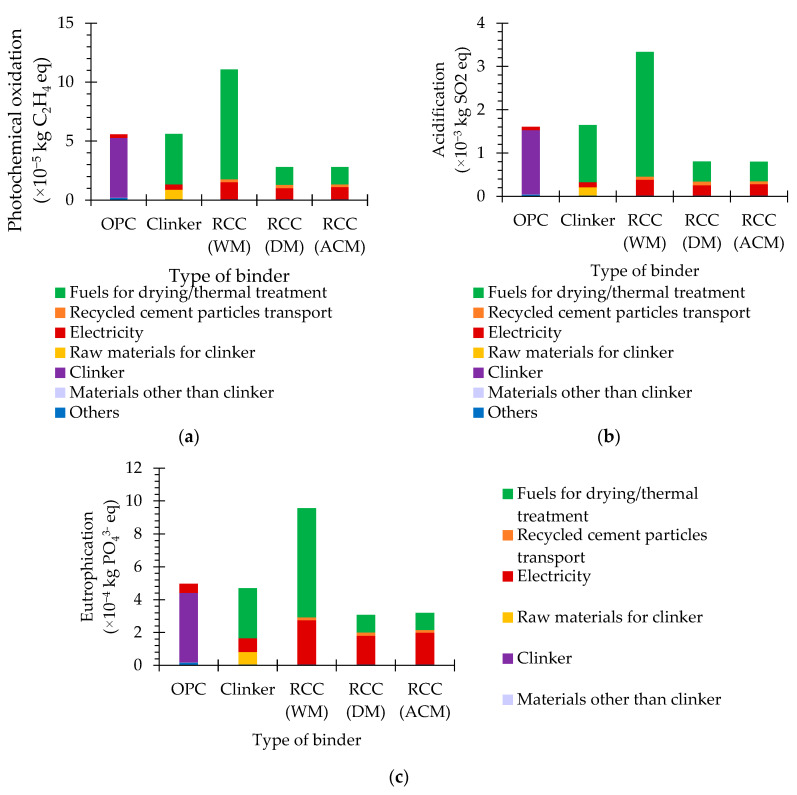
Photochemical oxidation (**a**), acidification (**b**) and eutrophication (**c**) potentials of different types of binder.

Similarly to other impact categories, the photochemical oxidation, acidification and eutrophication potentials of OPC were mainly affected by its clinker content, which was mostly governed by fuel consumption and, less meaningfully, by raw material and electricity consumption (Figure 6). As mentioned, RCC has no raw material consumption and the fuel needs for thermal activation are considerably lower than those of clinker. Therefore, the influence of these parameters in this impact category is not as expressive. On the other hand, RCC production involved a significantly higher amount of electricity, and in RCC WM, of fuel, than that of clinker.

Overall, depending on the separation method, the photochemical-oxidation, acidification and eutrophication potentials of RCC are about 50–199% (Figure 6a, Table 1), 50–208% (Figure 6b, Table 1) and 62–192% (Figure 6c, Table 1) of those of OPC, respectively.

The photochemical oxidation, acidification and eutrophication potentials of RCC DM and RCC ACM are about 25% (Figure 6a, Table 1), 24% (Figure 6b, Table 1) and 32–33% (Figure 6c, Table 1) of those of RCC WM, due to the lower electricity and fuel needs for the liberation and separation process of the cement paste particles from the concrete waste. Moreover, the photochemical oxidation, acidification and eutrophication potentials of RCC ACM are about 100% (Figure 6a, Table 1), 99% (Figure 6b, Table 1) and 104% (Figure 6c, Table 1) of those of RCC DM, respectively. Altogether, RCC DM and RCC ACM have a positive contribution tn the reduction in the photochemical depletion, acidification and eutrophication potentials compared to OPC.

## 6. Correction of RCC Life Cycle Assessment

As mentioned, the different production methods affected the cement paste content of the separated material (Section 2). Assuming that the thermal activation efficiency is constant, regardless of the level of purity of the material, this implies that the portion of reactive components in the RCC varies with the production method. Thus, for a more accurate comparison between these binders (RCC WM, RCC DM and RCC ACM), the LCA results of the RCC for the various methods were corrected by dividing them by the corresponding degree of purity of the binder (Table 2).

Overall, RCC ACM displays the lowest impact potential of the three RCCs. The abiotic depletion potentials of RCC ACM are about 51% and 73% (materials, Table 2) and 26% and 78% (fuels, Table 2) of those of RCC WM and RCC DM, respectively.

The global warming potential of RCC ACM is about 22% and 79% of those of RCC WM and RCC DM, respectively (Table 2). The ozone layer depletion potential of RCC ACM is about 30% and 78% of those of RCC WM and RCC DM, respectively (Table 2). The human toxicity and freshwater aquatic, marine aquatic and terrestrial ecotoxicity potentials of RCC ACM are about 42% and 80%, 45% and 84%, 45% and 84% and 21% and 79% of those of RCC WM and RCC DM, respectively (Table 2).

Finally, the photochemical oxidation, acidification and eutrophication potentials of RCC ACM are about 24 and 79%, 23% and 79% and 32 and 82% of those of RCC WM and RCC DM, respectively (Table 2).

These results can be explained by the fact that RCC ACM does not require the use of fuels and needs considerably less electricity than RCC WM during the liberation and separation phases. Moreover, the purity of RCC ACM is similar to that of RCC WM. On the other hand, despite the fact that RCC ACM needs slightly more electricity than RCC DM, the purity of RCC ACM was substantially higher than that of RCC DM.

## 7. Conclusions

This study aimed to demonstrate the potential contribution of RCC to the reduction in the environmental impacts of the cement industry, by means of a comparative life cycle assessment of different production methods of this binder and OPC. The RCC production processes’ performance were estimated using the approaches developed in Sousa and Bogas [35] and Sousa et al. [36]. In this study, the liberation and separation stage was considered to take place in the existing construction and demolition waste treatment facilities and the thermal activation to take place in the existing cement plants.

Overall, RCC WM did not turn out to be a good alternative to OPC, essentially owed to the amount of fuel and electricity required for washing and drying the particles before the magnetic separation. Nonetheless, as electricity production has been evolving towards cleaner renewable energy methods, the impact of electricity should decrease over time, closing the gap between RCC WM and OPC.

On the other hand, RCC DM and RCC ACM proved to be promising alternatives to RCC WM and OPC, with a relevant reduction in all impact categories. This difference should increase over time, with the improvement of the electricity production methods. In fact, the lower temperature required for the thermal treatment of RCC compared with the OPC production makes the replacement of fossil fuels with electricity more viable.

When RCC DM and RCC ACM were compared as a function of the cement content within the binder, RCC ACM displayed the lowest environmental impacts.

The results in this research are limited in two major aspects, one favourable and one unfavourable towards RCC: (i) the benefits from diverting concrete waste from landfilling and the high-quality fine aggregates generated in the RCC production process were not accounted for and (ii) the comparison between RCC and OPC did not account for the performance differences between these binders. The research on RCC produced using the methods presented in this study is still undergoing and the full understanding of the technical characteristics of the new material still requires further research (e.g., mechanical and durability performance). However, the published research results indicate the potential viability of RCC to be used as a partial replacement for OPC in various applications (e.g., concrete, mortar). This gains additional importance considering that some traditional products used nowadays are becoming scarcer. In particular, the reduction in the use of coal for electricity production is reducing the amount of fly ash generated. Furthermore, the amount of RCC that could be produced from the concrete waste generated annually would not meet the demand for cement in the same time frame.

## Figures and Tables

**Table 1:** Results of the life cycle assessment of different types of binder, according to each impact category.

Impact Category	Unit	OPC	Clinker	RCC (WM)	RCC (DM)	RCC (ACM)
Abiotic depletion (materials)	×10^−6^ kg Sb eq	2.20	1.55	1.55	0.90	0.83
Abiotic depletion (fossil fuels)	MJ	2.88	2.94	6.15	1.71	1.68
Global warming	kg CO_2_ eq	0.86	0.93	0.83	0.19	0.19
Ozone layer depletion	×10^−8^ kg CFC-11 eq	2.53	2.54	5.30	1.67	1.64
Human toxicity	×10^−3^ kg 1.4-DB eq	132.24	89.83	138.31	60.27	61.04
Freshwater aquatic ecotoxicity	×10^−3^ kg 1.4-DB eq	97.82	67.96	116.46	51.85	54.70
Marine aquatic ecotoxicity	×10^−3^ kg 1.4-DB eq	199,289	153,163	285,242	126,204	134,016
Terrestrial ecotoxicity	×10^−3^ kg 1.4-DB eq	1.28	1.32	2.75	0.60	0.60
Photochemical oxidation	×10^−5^ kg C_2_H_4_ eq	5.57	5.61	11.07	2.80	2.80
Acidification	×10^−3^ kg SO_2_ eq	1.61	1.65	3.33	0.81	0.80
Eutrophication	×10^−4^ kg PO_4_^3−^ eq	4.97	4.69	9.56	3.08	3.19

**Table 2:** Results of the life cycle assessment of different RCC production methods, according to each impact category.

Impact Category	Unit	RCC (WM)	RCC (DM)	RCC (ACM)
Abiotic depletion	×10^−6^ kg Sb eq	1.89	1.33	0.97
Abiotic depletion (fossil fuels)	MJ	7.50	2.52	1.97
Global warming	kg CO_2_ eq	1.01	0.28	0.22
Ozone layer depletion	×10^−8^ kg CFC-11 eq	6.46	2.46	1.92
Human toxicity	×10^−3^ kg 1.4-DB eq	168.59	88.91	71.52
Freshwater aquatic ecotoxicity	×10^−3^ kg 1.4-DB eq	141.95	76.50	64.09
Marine aquatic ecotoxicity	×10^−3^ kg 1.4-DB eq	347,693	186,190	157,028
Terrestrial ecotoxicity	×10^−3^ kg 1.4-DB eq	3.35	0.89	0.70
Photochemical oxidation	×10^−5^ kg C_2_H_4_ eq	13.50	4.13	3.28
Acidification	×10^−3^ kg SO_2_ eq	4.06	1.19	0.94
Eutrophication	×10^−4^ kg PO_4_^3−^ eq	11.65	4.54	3.74

## Data Availability

The data presented in this study will be made available upon reasonable request.
